# Supplementary figures and images for: The global burden of aortic aneurysm attributable to hypertension from 1990 to 2021: Current trends and projections for 2050
Source: PLoS One. 2025 Jul 18;20(7):e0327830. doi: 10.1371/journal.pone.0327830 (PMC12273936; doi:10.1371/journal.pone.0327830)

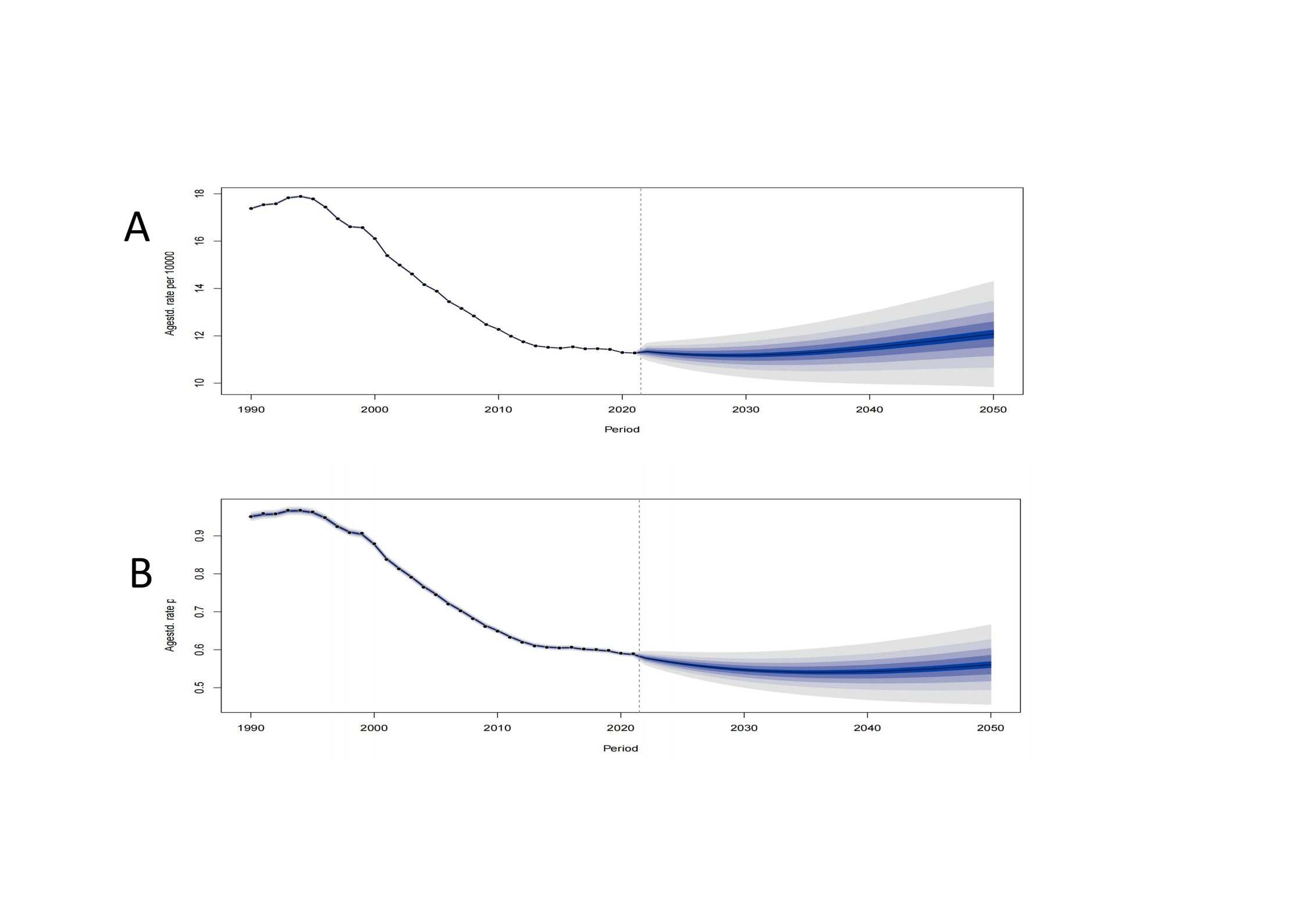

Supplement: S1 Fig — (TIF) [file pone.0327830.s001.tif]
